# Case of Strangulated Hernia Reduced by the Administration of Æther

**Published:** 1811-01

**Authors:** 

**Affiliations:** Bazas


*For the Medical and Physical Journal.*

*Case of strangulated Hernia reduced by the administration of Æther. Communicated by* Ardusset, M. D. *at Bazas, to the Editor of the Gazette de Sante, March* 1809.

DELOUMES, a hatter, aged about sixty years, of very spare habit, and bilious sanguine temperament, had been afflicted during fifteen years with inguinal hernia of the right side, which he reduced by the *taxis*, and kept up by means of a truss. This, however, he had discontinued about eighteen months, without any signs of the rupture being apparent.

In June, 1808, he rode a considerable distance on horseback in a very hot day; on his way he was seized with severe griping in the bowels, and a sharp pain in the right groin. He continued his journey, however, and returned home the next day without any further complaints than slight head-ach, and a painful sensation in the lower part of the abdomen.

The following day, June 17, whilst occupied in his shop, he was suddenly afflicted with a violent pain near the abdominal ring. Upon applying his hand to the part, he felt the tumour of the hernia, which he succeeded in returning within the abdomen. Two hours afterwards he ate a hearty dinner, and again entered his shop; but had scarcely commenced his usual employment, when the pain returned with a degree of intensity that scarcely permitted him to reach his chamber, and throw himself on his bed.

Whatever efforts he now made to reduce the hernia were unavailing. A common glyster was immediately thrown up, blood taken from the arm, and some hours afterwards he was placed in the warm bath, where he continued about an hour. The pain became easier, but the hardness and the volume of the tumour remained the same, and the following day the pain returned to an alarming degree. The patient was bled again, and put into the warm bath, which he could not bear beyond half an hour, although in the horizontal position. Upon being taken out of the bath, the swelling was covered with a large emollient poultice, which was renewed as often as it became cool. His drink was lemonade, and his nourishment chicken broth. He passed a restless night: the pain continued, and he was affected with hiccup and bilious vomiting. The pulse was small and frequent.

On the 19th, further assistance being deemed requisite, Dr. Ardusset was called in. His attempts at reduction proved ineffectual, and the symptoms became more threatening. He then then proposed the administration of a mixture of infusion of mint with æther, and applied compresses, dipped in æther, to the tumour. Soon after this practice was tried, the pain in the abdomen, the hiccup, and the vomiting, subsided; the swelling sensibly diminished, and by the next day totally disappeared;—since which the patient has had no return of the complaint.

